# Bayesian Spatio–Temporal Outbreak Detection for COVID-19 Mortality in South Africa: A Comparative Study of MCMC and Dynamic HMC Methods

**DOI:** 10.21203/rs.3.rs-9363716/v1

**Published:** 2026-04-21

**Authors:** SA. Darikwa, I. Maposa

**Affiliations:** 1 Division of Epidemiology and Biostatistics, Faculty of Medicine and Health Sciences, Stellenbosch University, Cape Town, South Africa

**Keywords:** Bayesian outbreak detection, Hamiltonian Monte Carlo, spatio-temporal modelling, COVID-19, South Africa, hidden Markov model, disease surveillance

## Abstract

**Background:**

Timely detection of localised COVID-19 surges is essential for targeting limited health resources, yet most routine surveillance algorithms ignore spatial dependence and many Bayesian spatio-temporal models are computationally demanding. Evidence on Hamiltonian Monte Carlo (HMC) performance for outbreak detection in low- and middle-income country (LMIC) settings remains limited. We applied a Bayesian spatio-temporal hidden Markov model (HMM) to South African COVID-19 hospital mortality, comparing data-augmented Markov chain Monte Carlo (MCMC) with dynamic HMC.

**Methods:**

We conducted a retrospective ecological time-series study of in-hospital COVID-19 deaths across 52 districts over 28 months (March 2020–June 2022), using national hospital surveillance linked to district-level health-system and population indicators. Death counts were modelled with a Poisson log-linear specification incorporating a smooth temporal trend, cyclic seasonality, spatial smoothing, and selected covariates, offset by expected deaths from admissions and a case-fatality ratio. Outbreaks were represented by a two-state HMM with latent indicators integrated out analytically and estimated via dynamic HMC in Stan. Eight candidate models were ranked using marginal likelihoods; the preferred model was re-fitted with both samplers to compare runtime, ESS, and convergence.

**Results:**

A spatial HMM with marginalised outbreak states was strongly favoured over non-outbreak and threshold-based alternatives. Posterior outbreak probabilities reproduced the four recognised national waves while revealing marked district-level heterogeneity, with early intense outbreaks in Western Cape and Gauteng districts and later peaks inland. Outbreaks were short-lived (mean around three months), and residual spatial risks indicated persistent excess mortality in the Eastern Cape and Free State. Dynamic HMC and MCMC yielded similar outbreak probability surfaces; however, HMC produced substantially larger ESS (approximately 5,000 versus 63) and near-ideal convergence, whereas MCMC showed poor mixing. ESS per second was similar, so HMC’s extra computation yielded more informative samples.

**Conclusions:**

A Bayesian spatio-temporal HMM fitted with dynamic HMC delivers coherent, spatially resolved outbreak probabilities and captures short-lived district-level mortality surges within broader national waves. Despite greater computational intensity, dynamic HMC offers superior mixing and convergence and is preferable for routine surveillance when adequate computing resources are available. The framework is transferable to other routinely collected surveillance data in South Africa and similar LMIC settings.

## 1 Background

Public health surveillance is defined as “the ongoing, systematic collection, analysis, and interpretation of outcome-specific data for use in the planning, implementation, and evaluation of public health practice” [28]. A critical requirement of surveillance systems is timeliness—the ability to produce actionable intelligence within intervention windows [10]. For rapidly evolving epidemics, this necessitates models that account for how infections spread across space and time while generating estimates quickly enough to inform response.

The COVID-19 pandemic, caused by the SARS-CoV-2 virus, has resulted in over 7 million confirmed deaths globally as of April 2024 [31]. This infectious respiratory disease spread rapidly across borders, overwhelming healthcare systems and exposing critical gaps in disease-surveillance capabilities worldwide. The pandemic’s defining features—rapid transmission, multiple waves of varying intensity, and marked geographic heterogeneity in timing and impact—make it an ideal case for evaluating advanced spatio-temporal surveillance methods. South Africa’s experience exemplified these challenges: COVID-19 mortality exhibited marked geographic heterogeneity with substantial variation across the 52 districts and spatial clustering patterns revealing distinct hot and cold spots of in-hospital deaths [21]. The timing and intensity of the pandemic varied substantially across all nine provinces, with multiple large waves occurring at different times and with different intensities across regions [32]. These spatio-temporal wave patterns underscore the need for methods that borrow strength across areas and time periods. We evaluate Bayesian approaches for district-level outbreak detection using COVID-19 hospital mortality—comparing traditional Markov chain Monte Carlo (MCMC) with dynamic Hamiltonian Monte Carlo (HMC)—to identify which method produces more accurate estimates in less time.

Classical surveillance algorithms such as the Farrington procedure and generalized linear models treat each time series independently, ignoring spatial connectivity and neighbourhood spillovers central to epidemic diffusion [11]. The COVID-19 pandemic made this limitation acute: outbreaks frequently moved from district to district, yet temporal-only models offered little early warning to neighbours experiencing initial increases. Foundational work in infectious-disease dynamics argues that spatial structure, seasonality, and population heterogeneity jointly shape observed incidence and mortality, and that models must capture these dominant features [2, 17].

Spatial Bayesian models arose to address these gaps. The space–time framework of Knorr-Held and Richardson [18] introduced switching between endemic and epidemic states across regions within a hierarchical model. Subsequent work extended this with spatial hidden Markov models (HMMs) that model when outbreaks start and how long they persist, while sharing information across neighbouring areas through conditional autoregressive (CAR) priors [27, 1]. More recent approaches added the ability to track multiple stages of transmission intensity, identify spatial clusters of high risk, and incorporate local factors such as healthcare capacity and population characteristics. These developments better capture how outbreaks spread geographically, how local conditions influence risk, and how epidemic waves unfold over time. The challenge of hierarchical spatio-temporal models is that they come with high computational cost. Traditional Bayesian implementations use MCMC methods that sample outbreak states and model parameters iteratively, requiring many iterations to explore complex relationships among spatial patterns, temporal trends, and outbreak indicators, leading to slow convergence—particularly for district-by-month HMMs with CAR structure [12, 20]. When outbreaks can escalate within days, these long runtimes limit practical utility.

Hamiltonian Monte Carlo (HMC) offers a gradient-based alternative that explores high-dimensional posteriors more efficiently than random-walk proposals. The dynamic HMC implementation in Stan (No-U-Turn Sampler, NUTS) adapts step sizes and path lengths automatically, improving effective sample sizes per unit time for hierarchical models [9, 13, 4]. A complementary innovation is to marginalise discrete HMM states by integrating them out analytically via forward filtering, so HMC samples only continuous parameters. Recent work integrated these advances into a comprehensive spatio-temporal outbreak-detection framework that combines CAR spatial effects, temporal random walks, seasonal components, and marginalised HMMs—demonstrating substantial reductions in computational burden while maintaining inferential accuracy [1].

However, this innovative framework has not been applied in low- and middle-income country settings. The performance of both the methodology and the comparative advantages of MCMC versus HMC remain unknown for operational contexts with data-quality challenges, resource constraints, and complex epidemiological profiles like those in South Africa, where high HIV prevalence, healthcare-infrastructure disparities, and socioeconomic inequalities shape epidemic dynamics [8, 24]. By benchmarking model fit, outbreak-detection performance, convergence behaviour, and runtime on district-level COVID-19 hospital mortality, we provide evidence-based guidance on which computational approach best supports real-time surveillance in South Africa and similar LMIC settings.

## 2 Methods

### 2.1 Study design and setting

We conducted a retrospective, ecological time–series study of district–month COVID-19 in-hospital mortality in South Africa. Analyses covered I=52 districts over T=28 months (March 2020–June 2022). The primary outcome was the district–month count of in-hospital COVID-19 deaths, $y_{it}$.

### 2.2 Data and preprocessing

District-level aggregated data on all laboratory-diagnosed COVID-19 hospital admissions and in-hospital deaths from March 2020 to June 2022 were assembled from the Daily Hospital Surveillance (DATCOV) database. Health-system and population-health indicators—including health-service coverage, hospital-bed density, health-worker density, HIV prevalence, and diabetes mellitus prevalence—were sourced from the District Health Barometer (DHB) 2019/20 and other public sources. The South African COVID-19 Vulnerability Index (SACVI) was included to measure district-level socioeconomic vulnerability. District boundaries were obtained from the Global Administrative Areas (GADM) database.

Records were aggregated to a complete 52 × 28 district–month lattice and linked using official district codes; district names were cleaned and standardised (ASCII folding, removal of administrative suffixes) to match the GADM shapefile. Limited missingness in the socioeconomic vulnerability index (sevuln) and the capacity-coverage index (capcover) was handled using multiple imputation by chained equations (MICE, m=5). All continuous covariates were standardised (mean 0, SD 1).

Candidate covariates were screened using pairwise correlations (|r|>0.70 threshold for exclusion), variance inflation factors (threshold 5), and 10-fold cross-validated Elastic Net regression, informed by prior literature on COVID-19 mortality determinants. The retained covariates—average age, ICU proportion, health-service coverage, private-sector share, and proportion male—were both statistically stable and epidemiologically meaningful. Hospital-bed density was excluded as a covariate because it was already incorporated through the expected-count offset.

Expected counts for the outbreak models were calculated as

$$E_{it}=\text{max}\left\{{\text{admissions}}_{it}\times \text{CFR},\varepsilon \right\},$$

with a small positive floor ε for numerical stability. The case–fatality ratio (CFR) was taken from national DATCOV hospital-surveillance estimates reported by the National Institute for Communicable Diseases (NICD) based on South African COVID-19 in-hospital mortality [23].

### 2.3 Modelling framework, computation, and comparison

We evaluated eight spatio-temporal models for outbreak detection (Table 1) using the DetectOutbreaks R package [1]. All models share a common Poisson log-linear backbone with a smooth temporal trend, seasonal effects, and spatial smoothing, ensuring a consistent baseline structure. Model 0 serves as the reference with no explicit outbreak term, capturing only the expected case counts without any outbreak-specific component. Models 1–7 extend the baseline model by incorporating a simple two-state (outbreak vs. non-outbreak) process that activates an additional elevation in risk during periods when an outbreak is occurring. These candidates differ in methodological approaches and assumptions about how outbreaks are detected. Models 1–4 employ classical (frequentist) outbreak-detection techniques built on top of the GLM fit. Farrington-type models (Models 1–3) use historical baseline windows and adjustments (with Model 3 adding overdispersion and trend correction), and Model 4 applies a CUSUM control chart to GLM residuals. In contrast, Models 5–7 take a Bayesian approach in which the model infers whether an outbreak is occurring by treating it as a hidden (latent) state estimated from the data, rather than using preset thresholds. Model 5 uses posterior predictive checks (Bayesian p-values) to identify anomalies, while Models 6 and 7 introduce explicit hidden outbreak indicators (a Bayesian change-point model via a two-state HMM, and a Bayesian CUSUM logic, respectively) that are inferred during model fitting.

For district i=1,…,I and month t=1,…,T, let $y_{it}$ denote observed in-hospital COVID-19 deaths and $E_{it}$ the expected count. Conditional on the mean $\mu_{it}$, counts follow

(1) $$y_{it}|\mu_{it}\sim \text{Poisson}\left(\mu_{it}\right),$$

(2) $$\text{log}\;\mu_{it}=\text{log}\;E_{it}+\alpha +r_{t}+s_{c(t)}+u_{i}+{\text{x}}_{i}^{⊤}\theta +\gamma_{it},$$

where α is an overall intercept; $r_{t}$ a smooth temporal trend (RW2); $s_{c(t)}$ a cyclic seasonal effect (RW1) by calendar month; $u_{i}$ a spatial random effect (ICAR); ${\text{x}}_{i}^{⊤}\theta$ district-level covariates; and $\gamma_{it}=z_{it}\beta_{0}$ the log-scale outbreak elevation, with $z_{it}\in \{0,1\}$ a latent outbreak indicator and $\beta_{0}$ the associated log-relative risk.

For each district, ${\left\{z_{it}\right\}}_{t=1}^{T}$ is a first-order Markov chain with transition probabilities $\gamma_{01}$ (baseline → outbreak) and $\gamma_{10}$ (outbreak → baseline). The latent states are integrated out analytically via the HMM forward algorithm, and district–month outbreak probabilities $P\left(z_{it}=1|y\right)$ are recovered by the forward–backward algorithm after sampling the continuous parameters.

Weakly informative priors were specified for fixed effects (α,θ), the outbreak elevation $\beta_{0}$, transition probabilities $\left(\gamma_{01},\gamma_{10}\right)$, and precision parameters. The temporal trend $r_{t}$ was modelled as a second-order random walk (RW2), the seasonal effects $s_{c(t)}$ as a cyclic first-order random walk (RW1), and the spatial effects $u_{i}$ as an intrinsic conditional autoregressive (ICAR) process defined on the district adjacency graph. Sum-to-zero constraints were imposed on $r_{t},\;s_{c(t)}$, and $u_{i}$ for identifiability, and non-centred parameterisations were used to improve sampling efficiency. Full hyperprior specifications are provided in Appendix A.

All eight candidate models were estimated using dynamic HMC (NUTS) via cmdstanr with four chains. We required split-$\hat{R}<1.05$, bulk and tail effective sample sizes >400 per parameter, and no divergent transitions. Posterior predictive checks assessed model fit. Models were ranked by marginal likelihoods estimated via importance sampling; full details are given in Appendix A.

To benchmark computation and confirm agreement, the top-ranked outbreak model was refit using the package’s data-augmented MCMC engine under identical data, priors, and specification; in that implementation, latent states $\left\{z_{it}\right\}$ are sampled via forward–backward algorithms while continuous parameters use Gibbs/Metropolis–Hastings updates. We compared effective sample size per second, log marginal likelihood $\left(\text{log}\;\hat{Z}\right)$, and outbreak-probability surfaces across samplers.

## 3 Results

### 3.1 Descriptive epidemiology of COVID-19 mortality

#### 3.1.1 Temporal dynamics

**Figure 1:**
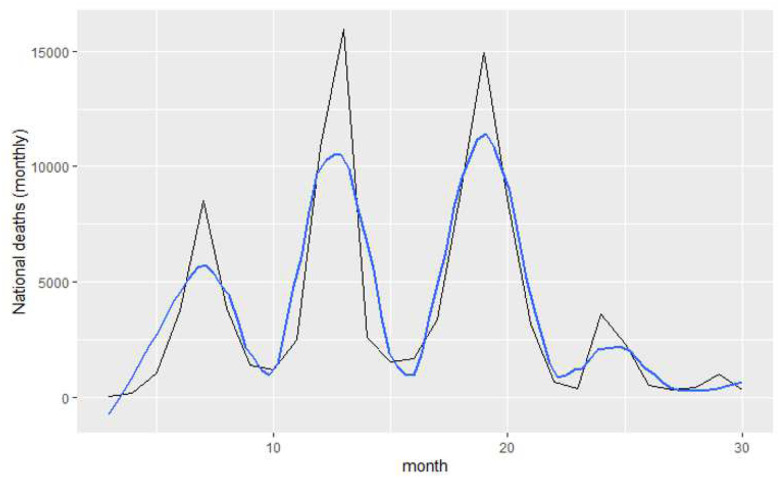
Monthly COVID-19 in-hospital deaths nationally (black), with LOESS smoother (blue), March 2020–June 2022. Peaks align with four major waves; post-wave mortality declines but remains above zero.

Figure 1 presents the national trajectory of monthly in-hospital COVID-19 deaths from March 2020 through June 2022, with four distinct epidemic waves clearly visible. Peak mortality occurred during the middle two waves, with monthly deaths exceeding ten thousand nationally, followed by progressively lower post-wave baselines.

The LOESS curve (Figure 1) reveals three patterns. First, wave peaks declined progressively after Wave 3. Second, deaths never returned to zero between waves, indicating persistent background mortality throughout the period. Third, the inter-peak interval shortened over time, from roughly six months (Waves 1–2) to three to four months (Waves 2–4).

#### 3.1.2 Spatio-temporal heterogeneity

**Figure 2:**
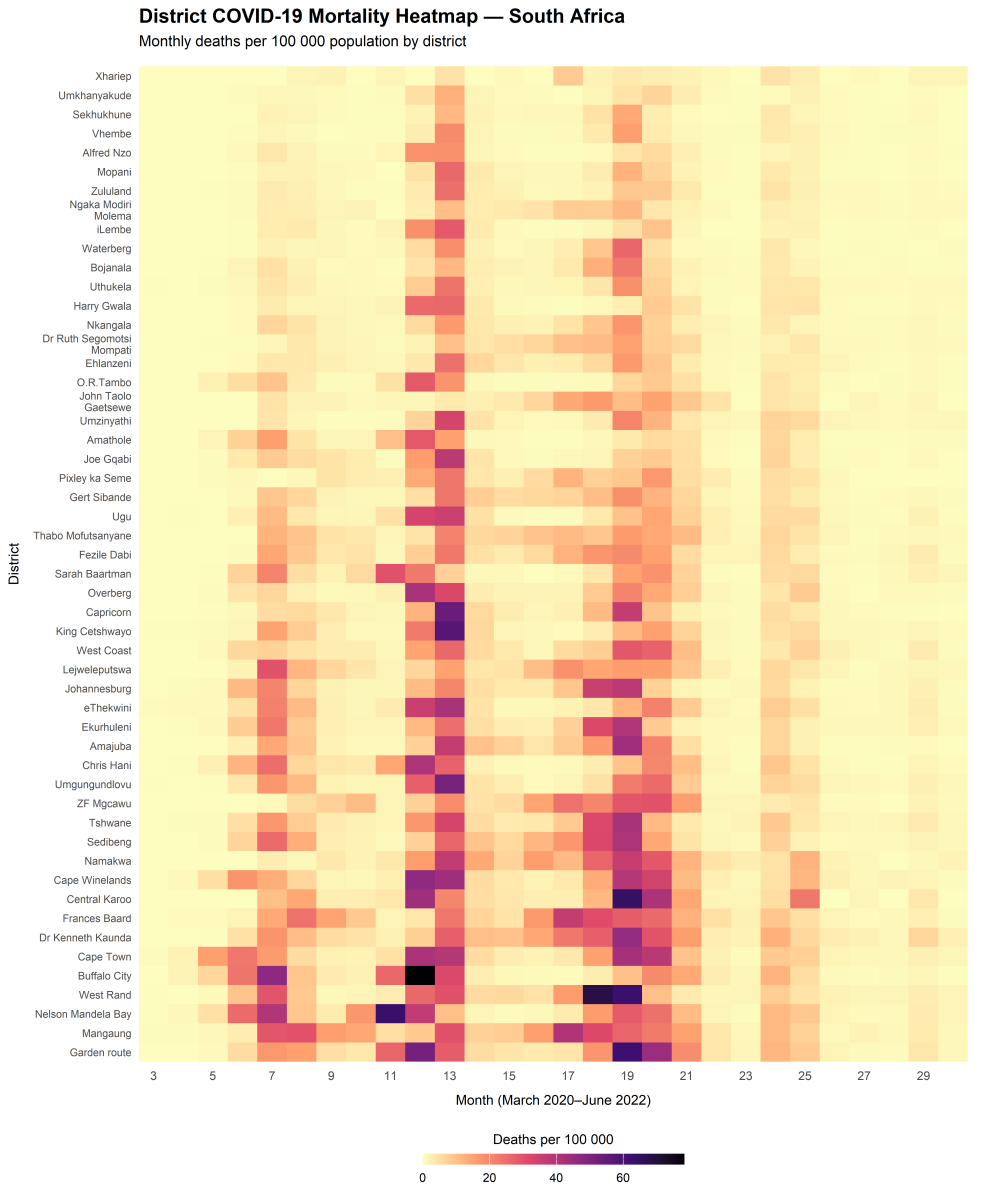
Heatmap of monthly COVID-19 deaths per 100,000 across 52 districts. Patterns reveal pronounced spatial and temporal heterogeneity in mortality burden.

Spatial variation in mortality timing and intensity (Figure 2) reveals a pandemic experienced very differently across districts. Western Cape metros such as Cape Town and Cape Winelands suffered early, steep Wave 1 peaks with monthly rates frequently exceeding 30–40 deaths per 100 000, consistent with rapid seeding in densely connected urban centres with high population mobility. In contrast, the West Coast district showed an earlier increase than many inland areas but with more modest Wave 1 peaks, remaining below approximately 20 deaths per 100 000. Several inland districts (for example Capricorn, ZF Mgcawu, Thabo Mofutsanyane) had minimal Wave 1 mortality but clearer rises in Waves 2–3, suggesting slower initial spread followed by substantial later burden.

Provincial patterns mirror this: in Gauteng, Johannesburg and Ekurhuleni display pronounced four-wave trajectories, whereas some surrounding districts show weaker early peaks but substantial mortality during later waves. In KwaZulu-Natal, eThekwini exhibits all four waves, whereas Zululand and uMkhanyakude show delayed early mortality with sharper peaks later. Overall, the heatmap indicates that the previous national wave summaries conceal meaningful district-level differences in timing and shifting hotspots over time.

**Figure 3:**
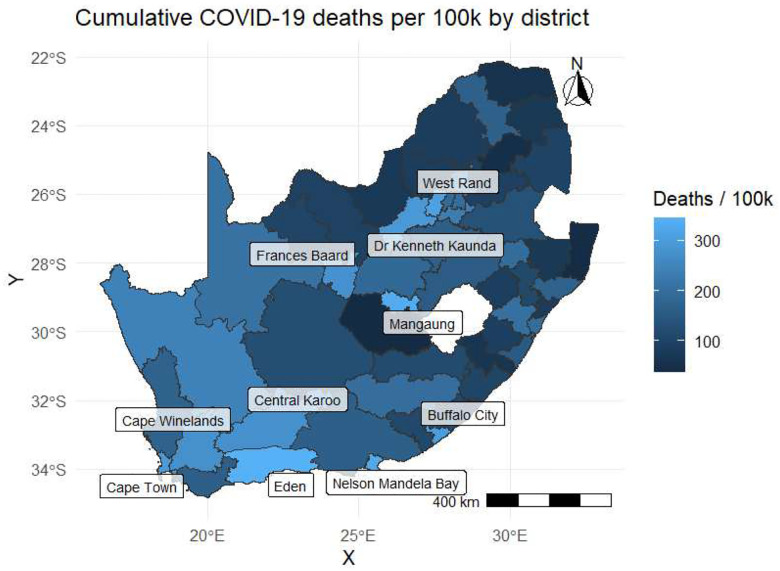
Cumulative COVID-19 deaths per 100,000 by district. Lighter shading indicates higher cumulative mortality; labels highlight example high-burden districts.

Cumulative mortality burden was geographically uneven (Figure 3). Higher-burden zones concentrated in coastal metros and adjacent districts—Cape Town, Cape Winelands, Nelson Mandela Bay, Buffalo City—with additional interior pockets including Frances Baard, Dr Kenneth Kaunda, Mangaung, and West Rand. Lower-burden areas predominated in the north and interior.

### 3.2 Spatial autocorrelation and clustering

#### 3.2.1 Global spatial autocorrelation

**Figure 4:**
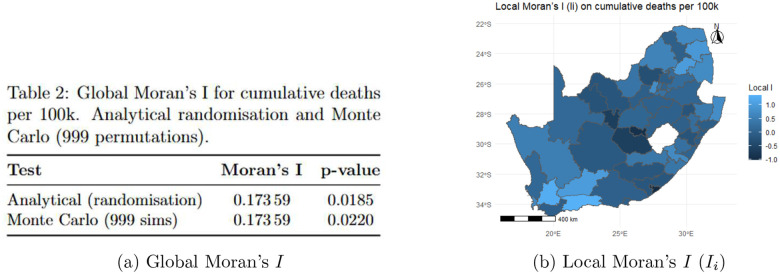
Spatial autocorrelation of cumulative deaths per 100,000. (A) Global Moran’s I indicates significant positive spatial clustering; (B) district-level $I_{i}$ values map local clustering intensity.

Cumulative deaths per 100,000 showed significant positive spatial autocorrelation (Moran’s I=0.174,p=0.019; Figure 4a), confirming that high-burden districts tend to neighbour other high-burden districts. The magnitude is moderate, indicating meaningful geographic clustering alongside substantial local variation.

#### 3.2.2 Local spatial autocorrelation

Local Moran’s $I_{i}$ (Figure 4b) identified the strongest clustering in the Western Cape coastal region, the Nelson Mandela Bay–Buffalo City corridor, and several Gauteng metropolitan districts. Several Eastern Cape interior and Free State districts had near-zero or negative $I_{i}$, indicating local mortality patterns diverging from their neighbours.

#### 3.2.3 Cluster classification

**Figure 5:**
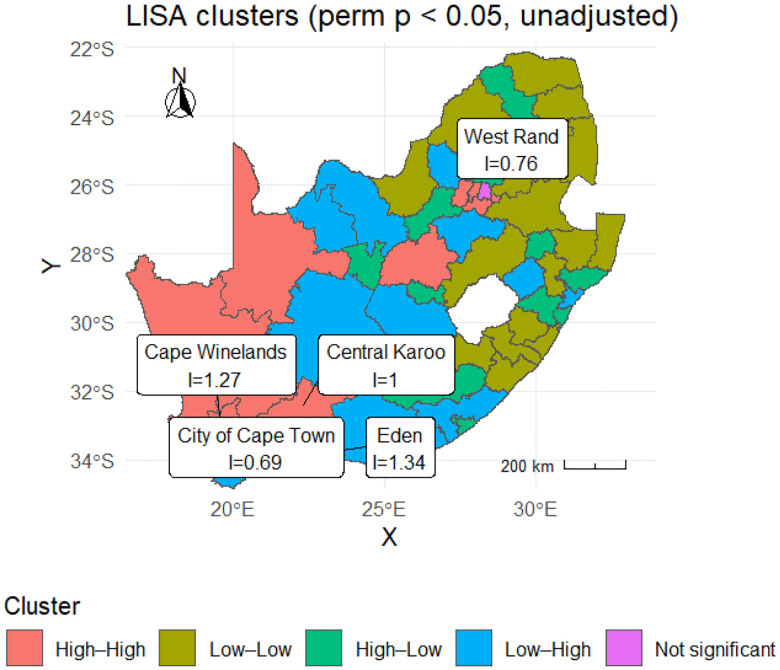
Local Indicators of Spatial Association (LISA) clusters for cumulative deaths per 100,000 (permutation p<0.05, unadjusted). Red: High–High hotspots; Green: Low–Low coldspots; Blue/Teal: spatial outliers; Grey: not significant.

Figure 5 refines this picture by classifying districts into four categories based on Local Indicators of Spatial Association (LISA) analysis at unadjusted p<0.05. High–High clusters identify statistically significant hotspots—districts with high mortality surrounded by high-mortality neighbours. These appear prominently in the Western Cape (City of Cape Town, Cape Winelands, Eden) and in selected interior districts such as Central Karoo and West Rand. Low–Low clusters mark significant coldspots where both the focal district and its neighbours experienced below-average mortality; these predominate in the northern interior (including much of Limpopo and parts of North West).

High–Low outliers are high-mortality districts bordered by low-mortality neighbours, potentially indicating localised drivers of excess risk that are not shared by adjacent areas. Low–High outliers represent the opposite pattern, where a relatively low-mortality district is surrounded by higher-mortality neighbours. These cluster patterns confirm that COVID-19 mortality in South Africa had a marked geographic structure, with coastal and selected interior districts forming significant hotspots, while other areas showed either protective clustering or outlying behaviour relative to their neighbours.

### 3.3 Model 6 fit, diagnostics, and key parameter estimates

Model 6 was the best-supported specification based on marginal likelihoods (Section 3.5), and we therefore focus on its fit, convergence diagnostics, and key parameters (Table 2). Convergence was excellent: all split-$\hat{R}$ values were 1.00, bulk and tail ESS exceeded 1,400 for every reported parameter, and no divergent transitions were observed. These diagnostics indicate reliable posterior exploration and motivate the use of Model 6 for subsequent inference.

The estimated HMM transition probabilities indicate rare outbreak onset and short persistence. The baseline-to-outbreak transition probability is $G_{12}=0.02$ (90% CrI: 0.01–0.03), while the outbreak-to-baseline probability is $G_{21}=0.32$ (90% CrI: 0.21–0.42). This implies an expected outbreak spell length of $1/G_{21}\approx 3.1$ months, consistent with the brief district-level mortality surges seen during national waves. The stationary outbreak probability, $\delta_{1}=G_{12}/\left(G_{12}+G_{21}\right)$, is approximately 0.06, confirming that the process spends most months in baseline conditions with intermittent epidemic departures.

Precision estimates indicated moderate spatial smoothing ($\kappa_{u}$ mean 1.10) and a highly right-skewed posterior for the seasonal precision $\kappa_{s}$, reflecting limited separability between seasonality and outbreak elevations during large waves.

#### 3.3.1 Spatial relative risks under Model 6

**Figure 6:**
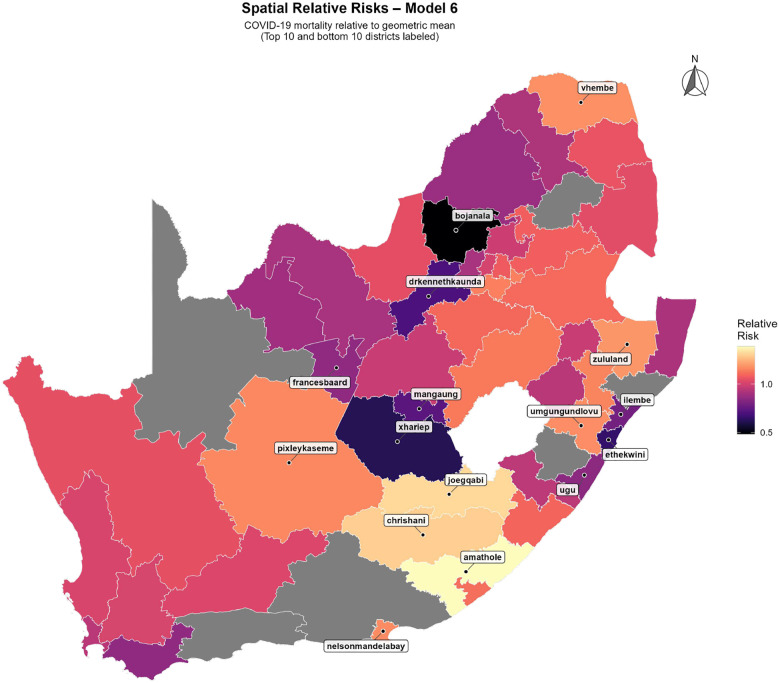
Spatial relative risks of COVID-19 mortality under Model 6, expressed relative to the national geometric mean. District labels show the ten highest- and ten lowest-risk areas.

Figure 6 displays the posterior spatial effects from Model 6 as district-level relative risks, conditional on expected counts, covariates, temporal trend, seasonality, and the latent outbreak process. Elevated residual risk persists in several Eastern Cape districts and parts of the Free State, echoing the high-burden zones seen in the descriptive maps and LISA hotspots. In contrast, multiple districts in the Western Cape, Northern Cape, and Limpopo show relative risks consistently below one, indicating lower-than-expected mortality once structural and epidemic components are accounted for. The close agreement between these residual risks and earlier cluster analyses suggests that the ICAR spatial term adequately captures remaining geographic structure in COVID-19 mortality.

### 3.4 Spatio-temporal outbreak detection

#### 3.4.1 Posterior outbreak probability heatmap

**Figure 7:**
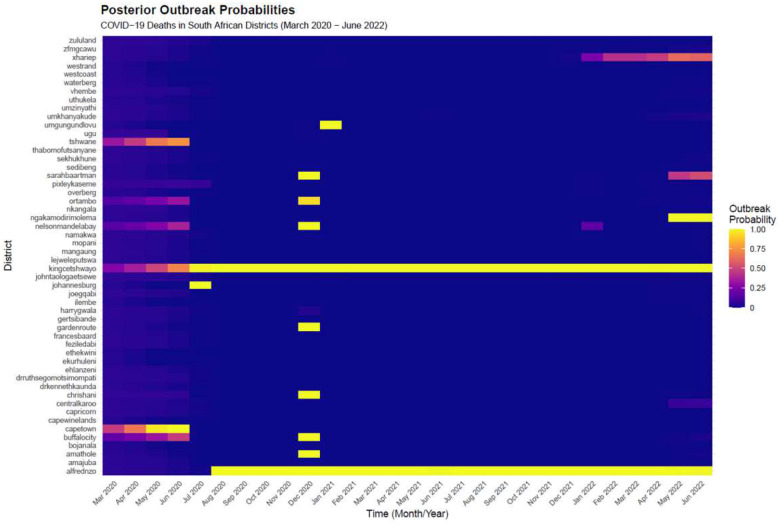
Posterior outbreak probabilities by district and month under Model 6. Bright cells indicate strong evidence of an outbreak; dark cells indicate baseline conditions.

Figure 7 displays the posterior outbreak probability $\text{Pr}\left(z_{it}=1|\right.\text{data})$ for each district and month under Model 6.

High outbreak probabilities align most clearly with Wave 1 (mid-2020), Wave 2 (late 2020/early 2021), and the Omicron-dominated Wave 4 (late 2021/early 2022). The Delta-dominated Wave 3 period (mid-2021) shows predominantly low outbreak probabilities across most districts. Between the major peaks, the surface is predominantly near-zero, indicating few inter-wave outbreak signals.

Geographic heterogeneity is pronounced. Several Western Cape districts—notably Cape Town—and Gauteng metros display intense Wave 1 outbreak signals, whereas many northern and inland districts show minimal Wave 1 probability but stronger signals in Waves 2–3. High posterior probability periods (> 0.8) typically lasted one to three consecutive months. Two districts—King Cetshwayo (KwaZulu-Natal) and Alfred Nzo (Eastern Cape)— displayed persistently elevated outbreak probabilities throughout the post-Wave 1 period, suggesting sustained local excess mortality warranting further investigation.

### 3.5 Model comparison

#### 3.5.1 Marginal likelihoods and Bayes factors

Bayes factors based on importance-sampling estimates of marginal likelihoods (Table 3) provide decisive evidence for including an explicit outbreak component. The Bayesian change-point specification (Model 6) attains by far the largest $\text{log}\;\overset{ˆ}{Z}$, yielding an implied posterior model probability effectively equal to 1.00 under equal prior model weights. The Farrington Flexible model (Model 2; $\text{log}\;\overset{ˆ}{Z}=-6465.36$) is the strongest non-Bayesian alternative but still far below Model 6 $\left(\Delta \text{log}\;\overset{ˆ}{Z}\approx 244\right)$. All in all, Bayes factors comparing Model 6 with the no-outbreak and threshold-based alternatives (Models 2–5, 7) are overwhelming, indicating that the hierarchical change-point parameterisation is the most strongly supported and the most reliable in practice.

### 3.6 Traditional MCMC versus dynamic HMC

To compare computational strategies, we re-fitted the best-supported outbreak model (Model 6) using the package’s data-augmented Gibbs/Metropolis sampler (“traditional MCMC”) and the dynamic Hamiltonian Monte Carlo implementation used for the main analysis (No-U-Turn sampler, “dynamic HMC”), under identical data, priors, and model specification.

Traditional MCMC completed in approximately 61 minutes, whereas dynamic HMC required about 5,030 minutes (83.8 hours), reflecting the additional cost of gradient evaluations and adaptation in the HMC run. Despite this, dynamic HMC produced many more effectively independent draws: for a representative spatial effect, the effective sample size (ESS) was about 5,000 under HMC versus only 63 under traditional MCMC, a nearly 80-fold increase. After normalising by runtime, the ESS per second was similar for both algorithms (approximately 0.017 ESS/sec, with HMC at about 0.96 times the traditional MCMC value), indicating that the extra HMC runtime is largely converted into additional effective samples rather than wasted computation. These runtime and efficiency comparisons are summarised in Figure 9.

**Figure 8:**
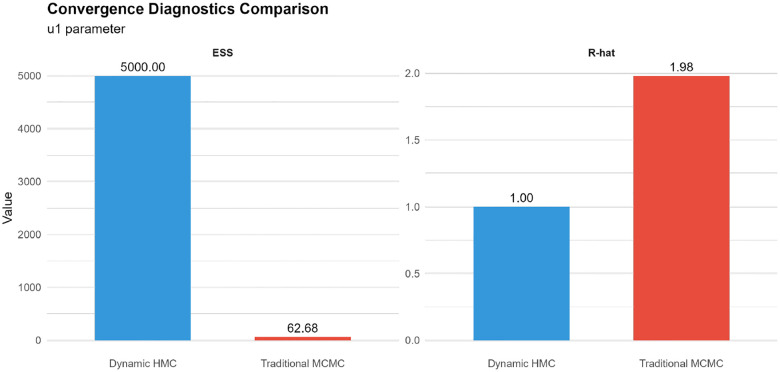
Convergence comparison between dynamic HMC and traditional MCMC for a representative Model 6 parameter: effective sample size (ESS, left panel) and Gelman–Rubin statistic ($\hat{R}$, right panel). Dynamic HMC attains much larger ESS and $\hat{R}$ values close to 1, indicating superior mixing and convergence.

Convergence diagnostics clearly favoured dynamic HMC (Figure 8). For the same parameter, the split $\hat{R}$ statistic was close to 2.0 under traditional MCMC, signalling substantial betweenchain disagreement and lack of convergence, whereas dynamic HMC achieved $\hat{R}\approx 1.00$ with no divergent transitions. Autocorrelation at lag 1 was very high for traditional MCMC (around 0.99), indicating strong random-walk behaviour and slow mixing, while dynamic HMC exhibited negligible autocorrelation and well-mixed trace plots. These diagnostics suggest that excessively longer or more heavily thinned traditional MCMC runs would be required to reach the same level of posterior exploration.

## 4 Discussion

Our study applied a Bayesian spatio-temporal hidden Markov model to detect COVID-19 mortality outbreaks across 52 South African districts from March 2020 to June 2022. Three main conclusions emerge. First, the spatial change-point specification (Model 6) was strongly supported over alternative outbreak formulations and classical threshold-based comparators, highlighting the value of explicitly modelling space, time, and latent outbreak states. Second, dynamic Hamiltonian Monte Carlo (HMC) delivered substantially better posterior exploration than traditional data-augmented MCMC. Third, the inferred district-level outbreak probabilities revealed marked heterogeneity that is obscured by national summaries and simple province-level wave descriptions.

The superiority of Model 6 over non-spatial and non-HMM alternatives is consistent with the theoretical understanding that infectious diseases spread through spatially connected populations [2, 17]. The moderate but significant spatial autocorrelation observed in cumulative mortality and the LISA patterns (Section 3.3; Figures 4–5) show that high-burden districts tend to cluster geographically, particularly in Western Cape and Gauteng metros, as also reported in previous work on South African COVID-19 mortality [21, 32]. At the same time, the presence of spatial outliers and districts with divergent local Moran’s $I_{i}$ values indicates that geographic proximity alone does not explain the observed patterns, supporting our inclusion of district-level random effects and covariates capturing health-system capacity and sociodemographic contexts.

**Figure 9:**
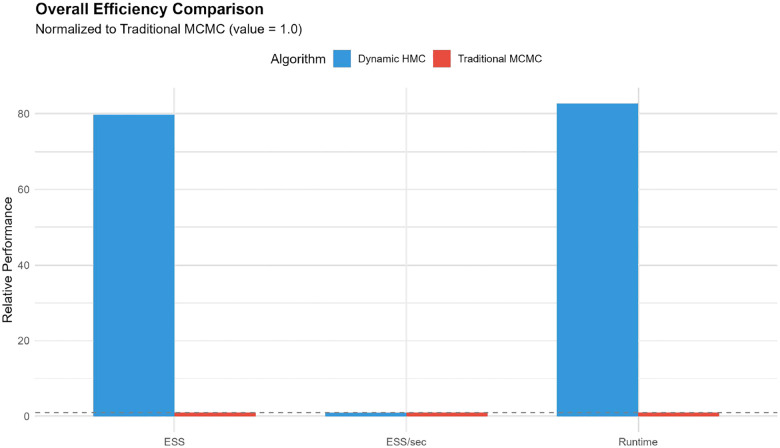
Summary of sampling efficiency for dynamic HMC and traditional MCMC under Model 6: total effective sample size (ESS), ESS per second, and wall-clock runtime. Dynamic HMC yields many more effective samples overall, with similar ESS per second but greater total computation time.

The comparison of traditional MCMC and dynamic HMC (Section 3.6; Figures 8–9) demonstrates that, for this class of hierarchical spatio-temporal models, gradient-based methods are far more reliable for routine surveillance. HMC produced order-of-magnitude gains in effective sample size and near-ideal convergence diagnostics, whereas traditional MCMC exhibited poor mixing and $\hat{R}$ values well above 1.1 despite long runs and thinning. When normalised by wall-clock time, both methods achieved similar effective samples per second, implying that HMC’s additional runtime translated directly into more informative posterior draws rather than being lost to tuning or random-walk behaviour. In practice, this suggests that, where computational resources allow, dynamic HMC should be preferred for operational deployment of such models; traditional MCMC is better reserved for quick prototyping or settings in which gradient-based software is unavailable [9, 4, 1].

The posterior outbreak probability surface adds a dynamic perspective to the descriptive epidemiology. While national series show four clear waves, modelled outbreak probabilities reveal that these waves were not experienced uniformly. Some Western Cape and Gauteng metros showed intense early Wave 1 outbreaks, whereas many inland districts displayed minimal early signals but pronounced Wave 2 and Wave 3 activity, echoing earlier descriptive analyses [32]. Two districts—King Cetshwayo and Alfred Nzo—showed persistently elevated outbreak probabilities beyond the national wave pattern, suggesting sustained local excess mortality warranting further investigation. The relatively muted Wave 3 signal reflects the model’s use of a time-varying baseline: after two large waves, the fitted baseline shifts upward, so that Deltaperiod mortality—though substantial—is less anomalous in relative terms. This is consistent with standard surveillance practice, which targets excess beyond a dynamic expected level rather than flagging all high-mortality periods [33, 34].

These results have several implications for public health surveillance in South Africa and similar low- and middle-income country settings. First, routine monitoring systems should incorporate spatial structure and not treat districts as independent time series. Posterior outbreak probabilities provide a principled way to communicate uncertainty: a threshold such as $\text{Pr}\left(z_{it}=1|\right.\text{data})>0.8$ could operationally flag district-months warranting investigation or resource reallocation, while moderate probabilities (0.4–0.7) may signal transitional periods meriting heightened surveillance. Second, because the estimated mean outbreak duration is approximately three months, early-warning systems should ideally operate at sub-monthly resolution if such data are available. Third, the framework requires only routinely collected counts, basic district boundaries, and a modest set of covariates, making it implementable by national institutes with standard statistical infrastructure.

Several limitations should be noted. First, we analysed in-hospital deaths, which occur 2–4 weeks after community transmission begins [7]. Using case data or symptom reports instead would allow earlier outbreak detection, but would also produce more false alarms. Second, we assumed a constant national case-fatality ratio, whereas time-varying severity due to emerging variants or vaccination could introduce confounding. Future work could incorporate dynamic severity estimates from sentinel surveillance. Third, queen contiguity may not fully capture functional connectivity in dense urban areas; gravity-model weights based on mobility data could refine spatial structure, though data availability in LMIC contexts remains a challenge. Fourth, the binary outbreak classification (yes/no) cannot distinguish between mild and severe outbreaks. A three-state model could capture these intensity differences and improve early warning, but would increase model complexity. Finally, because we analysed historical data retrospectively, we cannot verify whether the model would have detected outbreaks quickly enough in real-time. Prospective evaluation with incoming data is needed to validate alert timing [15, 14].

## 5 Conclusion

We developed and validated a Bayesian spatio-temporal outbreak detection pipeline for district-level COVID-19 mortality surveillance in South Africa. The best-supported model combined intrinsic conditional autoregressive spatial smoothing, flexible temporal baselines, and a two-state hidden Markov process fitted via dynamic Hamiltonian Monte Carlo. This approach produced coherent outbreak probabilities that matched national wave timing, revealed extensive district-level heterogeneity, and captured residual spatial risks beyond observed covariates.

The computational comparison demonstrated that dynamic HMC provides superior convergence and mixing compared to traditional data-augmented MCMC, with comparable efficiency when normalised by runtime. For routine public health surveillance applications, dynamic HMC is therefore the preferred estimation strategy when sufficient computing resources are available.

Some recommendations emanate from our study to strengthen infectious disease surveillance in South Africa and similar settings. First, explicitly model spatial dependencies since treating districts as independent units sacrifices both estimation precision and a realistic representation of how infections spread between neighbouring districts. Second, communicate outbreak uncertainty transparently through posterior probabilities rather than binary classifications, enabling decision-makers to match their response to the level of risk. Third, invest in computational infrastructure for gradient-based Bayesian methods, as the gains in convergence reliability justify the additional computational cost.

A natural direction for future work is to compare dynamic HMC with Integrated Nested Laplace Approximation (INLA) for fitting similar spatio-temporal outbreak models. Such head-to-head evaluations would clarify the trade-offs between fully sampled posterior inference and deterministic approximations in terms of accuracy, scalability, and turnaround time for routine surveillance. The framework presented here is readily transferable to other routinely collected surveillance outcomes and to similar low- and middle-income country contexts, provided district-level counts and basic geographic information are available.

## Supplementary Material

1

## Figures and Tables

**Table 1:** Summary of candidate models available via DetectOutbreaks::infer().

Model	Family	Key features
0	Baseline	Poisson GLM without an explicit outbreak component (reference).
1	Farrington (1996)	GLM with historical reference window for excess detection.
2	Farrington Flexible	Farrington with spline/seasonal flexibility to capture periodicity.
3	Improved Farrington	Adds overdispersion and trend adjustments to reduce false alarms.
4	CUSUM on GLM residuals	Control chart on residuals to flag sustained positive shifts.
5	Bayesian predictive p-value	Posterior-predictive anomaly scoring from fitted GLM.
6	Bayesian change-point (HMM)	Hierarchical model with explicit latent outbreak state; HMC fit.
7	Bayesian CUSUM	CUSUM logic embedded in a full Bayesian framework.

*Note:* All models were fit with the same offset/covariate setup for fair comparison.

**Table 2:** Model 6 posterior summaries and key sampler diagnostics.

Parameter	Mean	Median	SD	MAD	q5	q95	$\hat{R}$	ESS_bulk_	ESS_tail_
$G_{12}$	0.02	0.02	0.01	0.01	0.01	0.03	1.00	7634	16 376
$G_{21}$	0.32	0.31	0.06	0.06	0.21	0.42	1.00	11 020	20 461
$\delta_{1}$ (stationary outbreak prob.)	0.06	0.06	–	–	0.02	0.13	–	–	–
$\kappa_{u}$	1.10	1.08	0.23	0.22	0.75	1.49	1.00	22 509	36 850
$\kappa_{r}$	3.06	2.61	1.72	1.44	1.16	6.42	1.00	2502	12 649
$\kappa_{s}$	158.20	19.71	306.89	21.96	3.92	802.46	1.00	1484	7485

Notes: q5 and q95 define the central 90% credible interval. $G_{12}$ and $G_{21}$ are the monthly state-transition probabilities $\gamma_{01}=P(\text{endemic}\to \text{hyper-endemic})$ and $\gamma_{10}=P(\text{hyper-endemic}\to \text{endemic})$. The stationary outbreak probability $\delta_{1}=G_{12}/\left(G_{12}+G_{21}\right)$ is the long-run probability of being in the hyper-endemic (outbreak) state. $\kappa_{r},\;\kappa_{s}$, and $\kappa_{u}$ are precision parameters for the temporal trend, seasonal, and spatial random effects, respectively; larger κ corresponds to smoother (lower-variance) effects.

**Table 3:** Model comparison for the COVID-19 mortality application (equal prior model weights).

Model	Algorithm family	Log marginal likelihood	Posterior model probability
6	Bayesian change-point model	−6221.81	1.000
1	Farrington (1996)	−6244.03	0.000
2	Farrington Flexible	−6465.36	0.000
7	Bayesian CUSUM	−6564.04	0.000
4	CUSUM on GLM residuals	−6564.63	0.000
3	Improved Farrington	−6569.16	0.000
5	Bayesian predictive p-value	−6766.71	0.000

## Data Availability

Data analysed in this study are available upon reasonable request from the corresponding author.

## A Priors, identifiability, and computation

### Notation.

Indices i=1,…,I (districts), t=1,…,T (months), c(t)∈{1,…,12} (calendar month), with outcome $y_{it}$ and mean $\mu_{it}$. Offsets $E_{it}$ are the expected deaths defined in Section 2.2. Trend $\text{r}=\left(r_{1},\ldots ,r_{T}\right)$, seasonality $\text{s}=\left(s_{1},\ldots ,s_{12}\right)$, spatial effect $\text{u}=\left(u_{1},\ldots ,u_{I}\right)$, regression coefficients θ, intercept α, outbreak elevation $\beta_{0}$. Hidden state $z_{it}\in \{0,1\}$ with transition probabilities

$$\Gamma =\left(\begin{array}{cc} 1-\gamma_{01} & \gamma_{01}\\ \gamma_{10} & 1-\gamma_{10} \end{array}\right).$$

Precisions $\kappa_{r},\kappa_{s},\kappa_{u}$; standard deviations $\tau_{•}=\kappa_{•}^{-1/2}$. Stationary outbreak probability $\delta_{1}=\gamma_{01}/\left(\gamma_{01}+\gamma_{10}\right)$.

### A. 1 Baseline priors (fixed effects)

$$\alpha \sim 𝒩\left(0,5^{2}\right),\;\theta_{k}\sim 𝒩\left(0,2.5^{2}\right)\;\left(k=1,\ldots ,p\right),\;\beta_{0}\sim 𝒩\left(0,1.5^{2}\right).$$

These weakly informative scales regularise the intercept, covariate effects, and the outbreak log-relative risk while remaining compatible with the effect sizes expected from the literature.

### A. 2 Structured random effects

#### Temporal trend (RW2).

A second-order Gaussian random walk for smooth long-term variation:

$$r_{t}-2r_{t-1}+r_{t-2}\sim 𝒩\left(0,\kappa_{r}^{-1}\right)\;\left(t\geq 3\right),$$

with diffuse $\left(r_{1},r_{2}\right)$ and $\sum_{t}r_{t}=0$ for identifiability. Implementation uses a non-centred parameterisation $\text{r}=L_{r}\eta_{r},\;\eta_{r}\sim 𝒩(0,\text{I})$.

#### Seasonality (cyclic RW1).

A first-order cyclic GMRF over months with sum-to-zero constraint:

$$s_{c}-s_{c-1}\sim 𝒩\left(0,\kappa_{s}^{-1}\right),\;c=1,\ldots ,12,\;s_{0}\equiv s_{12},\;\sum_{c=1}^{12}s_{c}=0.$$

#### Spatial effect (ICAR).

An intrinsic CAR on the district graph:

$$\text{u}\sim \text{IGMRF}\left(\kappa_{u}R\right),\;\sum_{i=1}^{I}u_{i}=0,$$

where R is the structure matrix from queen contiguity ($R_{ii}=|n(i)|;R_{ij}=-1$ if j∈n(i)) [5, 6, 26]. A non-centred form $\text{u}=L_{u}\eta_{u},\;\eta_{u}\sim 𝒩(0,\text{I})$ is used.

### A. 3 Hyperpriors for precisions

We place weakly informative gamma priors on precisions,

$$\kappa_{r},\;\kappa_{s},\;\kappa_{u}\overset{\text{i.i.d.}}{\sim }\text{Gamma}(a,b),$$

with base a=1,b=0.01 (mean = 100, standard deviation ≈ 100). Sensitivity sets consider (a,b)∈{(0.5,0.005),(2,0.02)}. We also examine half-t priors on standard deviations $\tau_{•}\sim \text{Half-}t(\text{df}=4,\text{scale}=1)$ in robustness checks. These alternatives leave substantive conclusions unchanged.

### A. 4 Hidden Markov outbreak process

#### Initial state.

$$\pi =\text{Pr}\left(z_{i1}=1\right)\sim \text{Beta}(1,1)\;(uniform).$$

#### Transition probabilities.

$$\gamma_{01}\sim \text{Beta}(8,2),\;\gamma_{10}\sim \text{Beta}(8,2),$$

which mildly favour persistence while permitting rapid switching. The stationary outbreak probability and expected spell lengths follow from Γ:

$$\delta_{1}=\frac{\gamma_{01}}{\gamma_{01}+\gamma_{10}},\;E[\text{outbreak}\;\text{duration}]=\frac{1}{\gamma_{10}},\;E[\text{baseline}\;\text{duration}]=\frac{1}{\gamma_{01}}.$$

### A. 5 Marginal likelihood and model comparison

Because information criteria can be unreliable with discrete latent indicators, we rank candidates using Bayes factors based on importance-sampling estimates of the marginal likelihood Z=∫p(y∣θ)p(θ)dθ [16]. Let ${\left\{\theta^{\left(m\right)}\right\}}_{m=1}^{M}$ be posterior draws; we fit a heavy-tailed multivariate $t_{\nu }(\nu =3)$ proposal q(θ) to their mean/covariance and use

$$\hat{Z}=\frac{1}{M}\sum_{m=1}^{M}\frac{p\left(y|\theta^{\left(m\right)}\right)p\left(\theta^{\left(m\right)}\right)}{q\left(\theta^{\left(m\right)}\right)},$$

with log-sum-exp stabilisation and truncation of extreme weights when necessary. We report $\text{log}\;\hat{Z}$ with Monte Carlo error (batch means). The same estimator is applied to draws from both the HMC and data-augmented MCMC fits of the best model, enabling a like-for-like comparison of marginal evidence.

## List of abbreviations

CAR: Conditional autoregressive
CFR: Case-fatality ratio
COVID-19: Coronavirus disease 2019
DATCOV: Daily Hospital Surveillance (South African national COVID-19 hospital surveillance system)
DHB: District Health Barometer
ESS: Effective sample size
GADM: Global Administrative Areas
GIS: Geographic information system
GLM: Generalised linear model
HMC: Hamiltonian Monte Carlo
HMM: Hidden Markov model
ICAR: Intrinsic conditional autoregressive
LMIC: Low- and middle-income country
LISA: Local Indicators of Spatial Association
LOESS: Locally estimated scatterplot smoothing
LOO: Leave-one-out cross-validation
MCMC: Markov chain Monte Carlo
NICD: National Institute for Communicable Diseases
NUTS: No-U-Turn sampler
PCA: Principal component analysis
RW1: First-order random walk
RW2: Second-order random walk
SACVI: South African COVID-19 Vulnerability Index
SMR: Standardised mortality ratio
VIF: Variance inflation factor
