# Supplementary material for: Bayesian Spatio–Temporal Outbreak Detection for COVID-19 Mortality in South Africa: A Comparative Study of MCMC and Dynamic HMC Methods
Source: Res Sq. 2026 Apr 21:rs.3.rs-9363716. Preprint. [Version 1] doi: 10.21203/rs.3.rs-9363716/v1 (PMC13131857; doi:10.21203/rs.3.rs-9363716/v1)
Supplement: 1 [file NIHPPRS9363716V1-supplement-1.pdf]

B Supplementary Material S1: Prior sensitivity analysis

S1.1 Rationale and prior specifications

To evaluate the robustness of our main conclusions to prior choice, we performed a sensitivity analysis focused on the HMM transition probabilities, γ_{01} (baseline → outbreak) and γ_{10} (outbreak → baseline). The primary analysis specified γ_{01} and γ_{10} independently as Beta$(8, 2)$. To assess sensitivity, we considered two additional symmetric hyperprior specifications:

- **Weak prior**: Beta$(2, 2)$, approximately uniform on $(0, 1)$.
- **Strong prior**: Beta$(12, 2)$, placing more mass on high transition probabilities and thus shorter state durations.

For each specification we drew 100,000 samples from the joint prior for $(\gamma_{01}, \gamma_{10})$ and derived prior-implied quantities of interest. Summary measures are reported in Table S1.

S1.2 Prior-implied expectations

- **Base prior** (Beta$(8, 2)$): mean expected duration ≈ 1.29 months, median ≈ 1.22 months, $\mathrm{E}(\delta_1) \approx 0.50$.
- **Weak prior** (Beta$(2, 2)$): mean duration ≈ 2.98 months, median ≈ 2.00 months, $\mathrm{E}(\delta_1) \approx 0.50$.
- **Strong prior** (Beta$(12, 2)$): mean duration ≈ 1.18 months, median ≈ 1.14 months, $\mathrm{E}(\delta_1) \approx 0.50$.

All three specifications are symmetric in $(\gamma_{01}, \gamma_{10})$ and therefore centre the stationary outbreak probability at $\delta_1 = 0.5$, implying no prior preference for spending more or less time in the outbreak state overall.

Table S1: Prior-implied expectations for outbreak characteristics under three symmetric Beta hyperprior specifications for the HMM transition probabilities.

Prior scenario	Mean outbreak duration (months)	Median outbreak duration (months)	Mean δ_1
Base: Beta$(8, 2)$	1.29	1.22	0.50
Weak: Beta$(2, 2)$	2.98	2.00	0.50
Strong: Beta$(12, 2)$	1.18	1.14	0.50

Notes: Values are prior expectations based on 100,000 draws from each prior specification. The stationary outbreak probability δ_1 is centred at 0.5 under all three scenarios because the priors on γ_{01} and γ_{10} are symmetric.

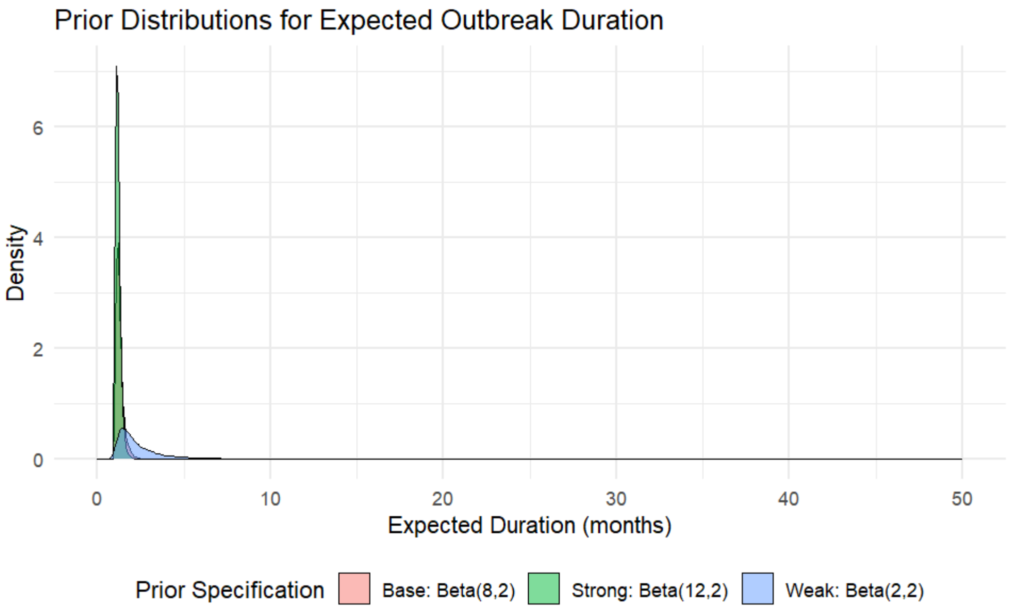

Figure S1: Prior distributions for the expected outbreak duration (months) under three hyper-prior specifications: Base Beta$(8, 2)$, Strong Beta$(12, 2)$, and Weak Beta$(2, 2)$.

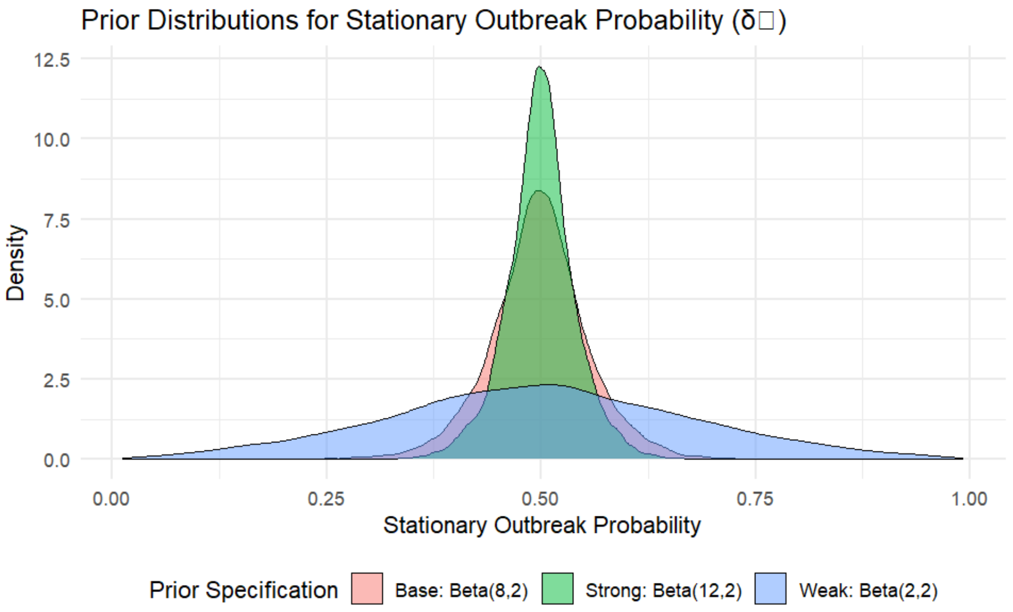

Figure S2: Prior distributions for the stationary outbreak probability δ_1 under the three hyper-prior specifications. All are centred near $\delta_1 = 0.5$ because of symmetry.

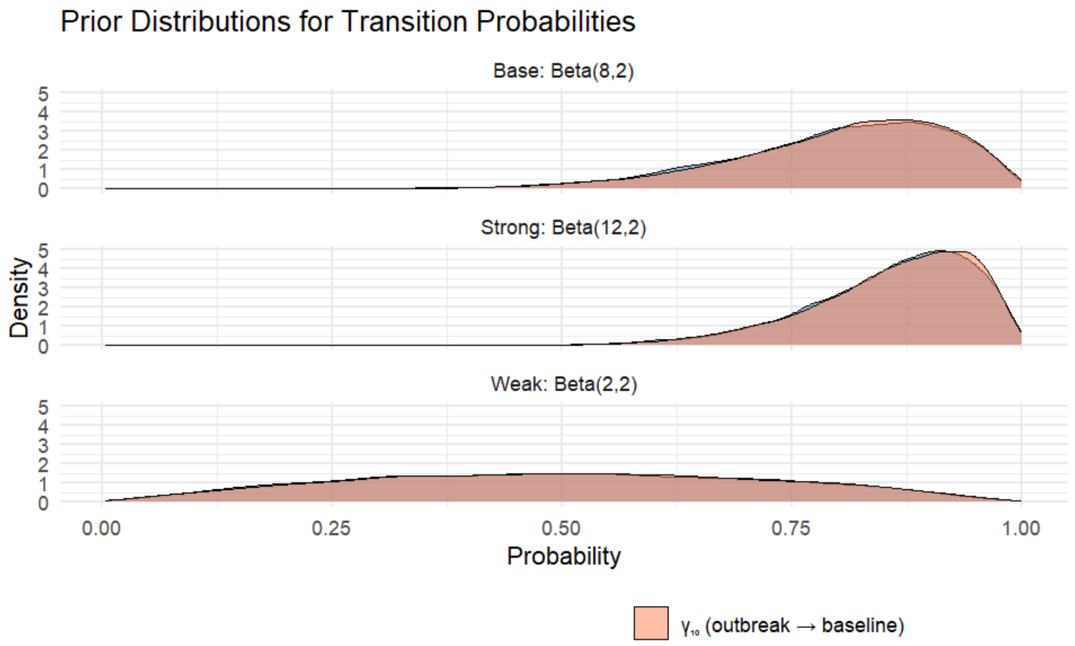

Figure S3: Prior densities for the transition probabilities γ_{01} and γ_{10} under the three symmetric Beta hyperpriors.

S1.3 Comparison with posterior estimates

The main analysis (Table 2) yielded:

$$\gamma_{01} \approx 0.02 \text{ (90\% CrI: } 0.01, 0.03), \qquad \gamma_{10} \approx 0.32 \text{ (90\% CrI: } 0.21, 0.42),$$

implying an expected outbreak duration of $1/\gamma_{10} \approx 3.1$ months and a stationary outbreak probability of $\delta_1 \approx 0.06$.

The posterior $\delta_1 \approx 0.06$ is far below the prior-implied value of 0.5 under all three scenarios, demonstrating substantial learning from the data. The posterior is also strongly asymmetric—outbreak onset is rare ($\gamma_{01} \approx 0.02$) while return to baseline is relatively rapid ($\gamma_{10} \approx 0.32$)—whereas all three priors treat these parameters symmetrically. This asymmetry is therefore driven entirely by the empirical mortality pattern, not by prior structure.

S1.4 Robustness of conclusions

The prior sensitivity analysis supports the robustness of our main conclusions. Posterior estimates for γ_{01}, γ_{10}, outbreak duration, and δ_1 differ markedly from prior-implied expectations under all three scenarios, indicating that the data are highly informative relative to the priors. Our key substantive conclusions—rare outbreak onset, short-to-moderate outbreak duration, and a low long-run outbreak probability—are compatible with the range of prior-implied behaviour across the three scenarios. Furthermore, the overwhelming advantage of Model 6 in log marginal likelihood relative to all alternatives reflects structural features that are not plausibly overturned by moderate variations in transition priors.

Reproducibility

Analyses were conducted in R ($\geq$4.3), `cmdstanr` (interface to Stan $\geq$2.37), and the `DetectOutbreaks` R package. Settings (chains, iterations, seeds, step-size target δ, `max_treedepth`) and all preprocessing and model scripts are archived with the study materials to ensure replicability.
